# Bias flow rate and ventilation efficiency during adult high-frequency oscillatory ventilation: a lung model study

**DOI:** 10.1186/s40635-018-0176-3

**Published:** 2018-04-19

**Authors:** Osamu Nagano, Tetsuya Yumoto, Atsunori Nishimatsu, Shunsuke Kanazawa, Takahisa Fujita, Sunao Asaba, Hideo Yamanouchi

**Affiliations:** 1 Department of Disaster and Emergency Medicine, Kochi University Medical School, 185-1, Kohasu, Oko-cho, Nankoku, Kochi 783-8505 Japan; 2 Advanced Emergency and Critical Care Medical Center, Okayama University Hospital, 2-5-1, Shikata-cho, Kita-ku, Okayama, 700-8558 Japan; 3 Center for Innovative and Translational Medicine, Kochi University Medical School, 185-1, Kohasu, Oko-cho, Nankoku, Kochi 783-8505 Japan

**Keywords:** High-frequency oscillatory ventilation (HFOV), Bias flow rate, Ventilation efficiency, Actual stroke volume

## Abstract

**Background:**

Bias flow (BF) is essential to maintain mean airway pressure (MAP) and to washout carbon dioxide (CO_2_) from the oscillator circuit during high-frequency oscillatory ventilation (HFOV). If the BF rate is inadequate, substantial CO_2_ rebreathing could occur and ventilation efficiency could worsen. With lower ventilation efficiency, the required stroke volume (SV) would increase in order to obtain the same alveolar ventilation with constant frequency. The aim of this study was to assess the effect of BF rate on ventilation efficiency during adult HFOV.

**Methods:**

The R100 oscillator (Metran, Japan) was connected to an original lung model internally equipped with a simulated bronchial tree. The actual SV was measured with a flow sensor placed at the Y-piece. Carbon dioxide (CO_2_) was continuously insufflated into the lung model ($\dot{\mathrm{V}}$CO_2_), and the partial pressure of CO_2_ (PCO_2_) in the lung model was monitored. Alveolar ventilation ($\dot{\mathrm{V}}$A) was estimated as $\dot{\mathrm{V}}$CO_2_ divided by the stabilized value of PCO_2_. $\dot{\mathrm{V}}$A was evaluated by setting SV from 80 to 180 mL (10 mL increments, *n* = 5) at a frequency of 8 Hz, a MAP of 25 cmH_2_O, and a BF of 10, 20, 30, and 40 L/min (study 1). Ventilation efficiency was calculated as $\dot{\mathrm{V}}$A divided by the actual minute volume. The experiment was also performed with an actual SV of 80, 100, and 120 mL and a BF from 10 to 60 L/min (10 L/min increments: study 2).

**Results:**

Study 1: With the same setting SV, the $\dot{\mathrm{V}}$A with a BF of 20 L/min or more was significantly higher than that with a BF of 10 L/min. Study 2: With the same actual SV, the $\dot{\mathrm{V}}$A and the ventilation efficiency with a BF of 30 L/min or more were significantly higher than those with a BF of 10 or 20 L/min.

**Conclusions:**

Increasing BF up to 30 L/min or more improved ventilation efficiency in the R100 oscillator.

**Electronic supplementary material:**

The online version of this article (10.1186/s40635-018-0176-3) contains supplementary material, which is available to authorized users.

## Background

Bias flow (BF) is essential to maintain mean airway pressure (MAP) and to washout carbon dioxide (CO_2_) from the oscillator circuit (“CO_2_ washout”) during high-frequency oscillatory ventilation (HFOV). If the BF rate is inadequate, substantial CO_2_ rebreathing could occur, and the resultant hypercapnia might become a problem [1, 2]. This situation increases wasted ventilation and worsens ventilation efficiency. With lower ventilation efficiency, the required stroke volume (SV) must increase in order to obtain the same alveolar ventilation with constant frequency.

The SensorMedics 3100B oscillator (CareFusion, Yorba Linda, CA, USA) has often been used with a BF of 30 or 40 L/min [3–5]. For the R100 oscillator (Metran Co. Ltd., Kawaguchi, Saitama, Japan), a BF of 20 to 40 L/min is recommended in Japan, although a BF of 30 L/min or more has been preferred in many institutions, especially in patients with spontaneous breathing. Some studies have investigated the effect of the BF rate [1, 2, 6, 7], although none have reported the exact effect of the BF rate on ventilation efficiency during adult HFOV. The aim of this study is to assess the effect of the BF rate on ventilation efficiency using the R100 oscillator.

## Methods

### Experimental setting

**Fig. 1:**
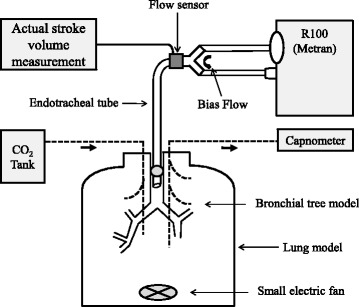
Schema of the experimental setting. Legend: For more information, see the text

The R100 oscillator was connected to the lung model via an angle-type connector and an endotracheal tube (ETT) with an internal diameter of 8.0 mm and a length of 30 cm (Fig. 1). A microelectromechanical systems mass flow sensor (Siargo FS6022B150, Siargo Ltd., Santa Clara, CA, USA) was placed between the angle-type connector and the Y-piece for the actual SV (aSV) measurement. The total dead space volume (VD) was approximately 110 mL, and the airway resistance was approximately 2.0 cmH_2_O/L/s. The common oscillator settings during the experiments were as follows: frequency of 8 Hz, MAP of 25 cmH_2_O, and fraction of inspired oxygen of 0.21. The inspiratory time was fixed at 50% in this oscillator. The heated humidifier was turned off.

### Measurement of actual stroke volume (aSV)

The aSV was measured with a prototype SV measurement system (Metran Co. Ltd., Kawaguchi, Saitama, Japan). In this system, the analogue flow signal was sampled at 200 Hz and digitally integrated to determine expiratory SV every second using a computer data acquisition system (LabView Ver. 14, National Instruments, Austin, TX, USA). The mean value of 60 data measurements for 1 min was calculated as the aSV.

### Lung model

The lung model consisted of a 20-L airtight rigid plastic container internally equipped with a simulated bronchial tree (KYOTO KAGAKU Co. Ltd., Kyoto, Japan) that had three to seven steps of bifurcations to 20 segmental bronchial branches (Fig. 1). The top of the ETT was located 3.5 cm from the carina. The total 20 L volume of the container accounts for an adiabatic static compliance of 19.3 mL/cmH_2_O (approximately equal to severe ARDS [8]) due to gas compression. The container had two ports for gas insufflation and for gas sampling. A small electrical fan was placed horizontally on the bottom to assist gas mixing in the container.

### Measurement of alveolar ventilation ($\dot{\mathrm{V}}$a)

CO_2_ was insufflated into the lung model at approximately 200 mL/min (float-type area flowmeter), and continuous gas sampling was performed from the lung model using a capnometer (Life Scope TR, NIHON KOHDEN Co., Tokyo, Japan) (Fig. 1). The gas sampling rate of the capnometer was set at 200 mL/min. The partial pressure of CO_2_ (PCO_2_; mmHg) was monitored by the capnometer, and the stabilized value was recorded. The actual minute volume of insufflated CO_2_ ($\dot{\mathrm{V}}$CO_2_; mL/min) was calculated by the measurement of PCO_2_ using the capnometer when it was mixed with oxygen at 5 L/min (fixed-type flowmeter). This was performed before starting the experiment and was confirmed every 1 to 2 h. The $\dot{\mathrm{V}}$A was estimated by applying the alveolar ventilation equation (PCO_2_ = 0.863 × $\dot{\mathrm{V}}$CO_2_/$\dot{\mathrm{V}}$A, PCO_2_ and $\dot{\mathrm{V}}$A: BTPS, $\dot{\mathrm{V}}$CO_2_: STPD), though the equation was rearranged and used as $\dot{\mathrm{V}}$A = $\dot{\mathrm{V}}$CO_2_/PCO_2_ because all experiments were done under room temperature and dry conditions.

Although the SV can be set at up to 205 mL with a frequency of 8 Hz in the R100 oscillator, the measurable range of the flow sensor was limited. Therefore, $\dot{\mathrm{V}}$A was evaluated with the SV setting (sSV) from 80 to 180 mL (10 mL increments) and with the BF from 10 to 40 L/min (10 L/min increments; study 1). Additionally, the ratio of $\dot{\mathrm{V}}$A to the actual minute ventilation volume (a$\dot{\mathrm{V}}$E; aSV/1000 × 8 × 60, L/min) was calculated ($\dot{\mathrm{V}}$A/a$\dot{\mathrm{V}}$E) as an index for ventilation efficiency. $\dot{\mathrm{V}}$A was also evaluated with the targeted aSV of 80, 100, and 120 mL and with the BF of 10 to 60 L/min (10 L/min increments; study 2). Because the BF rate can be set from 10 to 40 L/min in the R100 oscillator, air was added to the BF supply port using a float-type area flowmeter. To further examine the role of BF, the relationship between $\dot{\mathrm{V}}$A and a$\dot{\mathrm{V}}$E/BF was investigated in study 2.

Each experiment was conducted five times.

### Statistical analysis

The statistical analysis was performed with BellCurve for Excel ver. 2.02 (SSRI Co. Ltd., Tokyo, Japan) using one-way analysis of variance followed by Tukey’s test. *P* < 0.05 was considered statistically significant. Curve fitting was also performed by the same software.

## Results

### Study 1

**Fig. 2:**
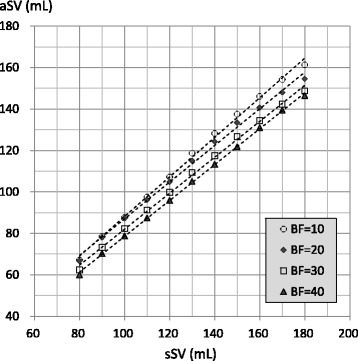
Relationship between setting stroke volume (sSV) and actual stroke volume (aSV). Legend: Marker indicates the mean value of aSV (*n* = 5). Standard deviation is not indicated. The results of statistical significance test are as follows: BF = 10 vs BF = 20, ns with sSV = 80–110, *P* < 0.05 with sSV = 120, *P* < 0.01 with sSV = 130–150, *P* < 0.001 with sSV = 160–180; BF = 10 or 20 vs BF = 30 or 40, *P* < 0.001 with all sSV; and BF = 30 vs BF = 40, *P* < 0.05 with sSV = 80 and 160–170, *P* < 0.01 with sSV = 90 and 130–150, *P* < 0.001 with sSV = 100–120, ns with sSV = 180. Dotted lines are the first-order approximations. The coefficient of correlations (*R*) and *P* values are as follows: BF = 10: *R* = 0.998, *P* < 0.001; BF = 20: *R* = 0.998, *P* < 0.001; BF = 30: *R* = 0.998, *P* < 0.001; and BF = 40: *R* = 0.999, *P* < 0.001

**Fig. 3:**
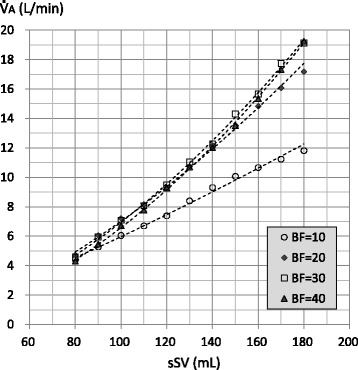
Relationship between setting stroke volume (sSV) and alveolar ventilation ($\dot{\mathrm{V}}$A). Legend: Marker indicates the sSV and mean value of $\dot{\mathrm{V}}$A (*n* = 5). Standard deviation is not indicated. The results of the statistical significance test are as follows: BF = 10 vs BF = 20, *P* < 0.001 with all sSV; BF = 10 vs BF = 30, *P* < 0.01 with sSV = 80, *P* < 0.001 with SV = 90–180; BF = 10 vs BF = 40, *P* < 0.05 with sSV = 80–90, *P* < 0.001 with sSV = 100–180; BF = 20 vs BF = 30, *P* < 0.05 with sSV = 80, 130, and 150, ns with sSV = 90–120 and 140, *P* < 0.01 with sSV = 160, *P* < 0.001 with sSV = 170–180; BF = 20 vs BF = 40, *P* < 0.001 with sSV = 80–100 and 170–180, *P* < 0.01 with sSV = 110, ns with sSV = 120–160; and BF = 30 vs BF = 40, *P* < 0.001 with sSV = 80–100, *P* < 0.05, with sSV = 110, 130, and 150, ns with sSV = 120, 140, and 160–180. Dotted curves are power approximations. The perfect powers (coefficient of correlation: *R*, *P* value) are as follows: BF = 10: 1.226 (*R* = 0.995, *P* < 0.001); BF = 20: 1.580 (*R* = 0.998, *P* < 0.001); BF = 30: 1.744 (*R* = 0.995, *P* < 0.001); and BF = 40: 1.819 (*R* = 0.994, *P* < 0.001)

Figure 2 shows the relationships between sSV and aSV at respective BF rates. The aSV was proportional to the sSV at all BF rates. Increasing the BF rate decreased the aSV at most settings. In all sSVs, the aSV with a BF of 30 or 40 L/min was significantly lower than that with a BF of 10 or 20 L/min (*P* < 0.001). The airway pressure amplitude (AMP) measured at the Y-piece (displayed on the panel of the R100 oscillator) showed a similar change (Additional file 1). Figure 3 shows the relationships between sSV and $\dot{\mathrm{V}}$A at respective BF rates. The $\dot{\mathrm{V}}$A was correlated to the power of sSV at all BF rates. The perfect powers were 1.226, 1.580, 1.744, and 1.819 with BFs of 10, 20, 30, and 40 L/min, respectively. In all sSVs, the $\dot{\mathrm{V}}$A with a BF of 20 L/min or more was significantly higher than the $\dot{\mathrm{V}}$A with a BF of 10 L/min (the *P* value was different for the sSV). The $\dot{\mathrm{V}}$A with a BF of 30 or 40 L/min was not significantly higher than the $\dot{\mathrm{V}}$A with a BF of 20 L/min in all sSVs. The $\dot{\mathrm{V}}$A with BFs of 30 and 40 L/min was not significantly different in all sSVs.

**Fig. 4:**
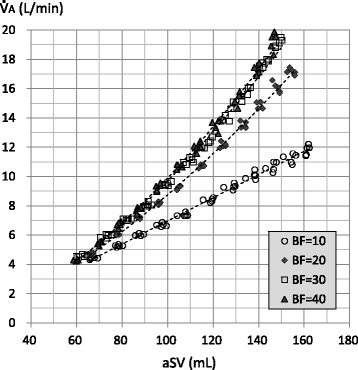
Relationship between actual stroke volume (aSV) and alveolar ventilation ($\dot{\mathrm{V}}$A). Legend: Markers indicate individual data of aSV and $\dot{\mathrm{V}}$A. Dotted curves are power approximations. The perfect powers (coefficient of correlation: *R*, *P* value) are as follows: BF = 10: 1.128 (*R* = 0.996, *P* < 0.001); BF = 20: 1.528 (*R* = 0.995, *P* < 0.001); BF = 30: 1.631 (*R* = 0.992, *P* < 0.001); and BF = 40: 1.664 (*R* = 0.992, *P* < 0.001)

Figure 4 shows the relationships between aSV and $\dot{\mathrm{V}}$A at respective BF rates (individual data). The $\dot{\mathrm{V}}$A was correlated with the power of the aSV at all BF rates. The perfect power were 1.128, 1.528, 1.631, and 1.664 with BFs of 10, 20, 30, and 40 L/min, respectively. It appeared that the $\dot{\mathrm{V}}$A increased with an increasing BF up to 30 L/min with an aSV of more than approximately 100 mL (a statistical analysis was not conducted).

**Fig. 5:**
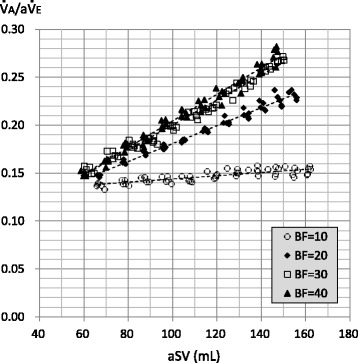
Relationship between actual stroke volume (aSV) and ventilation efficiency ($\dot{\mathrm{V}}$A/a$\dot{\mathrm{V}}$E). Legend: Markers indicate individual data of aSV and $\dot{\mathrm{V}}$A/a$\dot{\mathrm{V}}$E. Dotted lines are the first-order approximations. The coefficient of correlations (*R*) and *P* values are as follows: BF = 10: *R* = 0.824, *P* < 0.001; BF = 20: *R* = 0.989, *P* < 0.001; BF = 30: *R* = 0.990, *P* < 0.001; and BF = 40: *R* = 0.996, *P* < 0.001

Figure 5 shows the relationships between aSV and $\dot{\mathrm{V}}$A/a$\dot{\mathrm{V}}$E at respective BF rates (individual data). The $\dot{\mathrm{V}}$A/a$\dot{\mathrm{V}}$E was proportional to the aSV at all BF rates. It appeared that the $\dot{\mathrm{V}}$A/a$\dot{\mathrm{V}}$E increased with an increasing BF up to 30 L/min with an aSV of more than approximately 100 mL (a statistical analysis was not conducted).

### Study 2

**Fig. 6:**
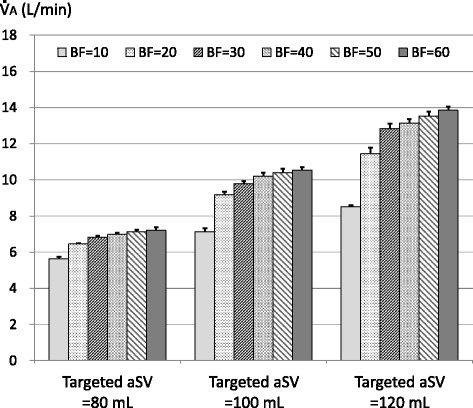
Alveolar ventilation ($\dot{\mathrm{V}}$A) measured with targeted actual stroke volume (aSV) of 80, 100, and 120 mL. Legend: Bar graph indicates mean $\dot{\mathrm{V}}$A (*n* = 5), and vertical bar indicates standard deviation. The results of statistical significance test are as follows: BF = 10 vs BF = 20–60: *P* < 0.001 with all aSV; BF = 20 vs BF = 30–60: *P* < 0.001 with all aSV; BF = 30 vs BF = 40: ns with aSV = 80 and 120, *P* < 0.05 with aSV = 100; BF = 30 vs BF = 50: *P* < 0.01 with aSV = 80 and 120, *P* < 0.001 with aSV = 100; BF = 30 vs BF = 60: *P* < 0.001 with all aSV; BF = 40 vs BF = 50: ns with all aSV; BF = 40 vs BF = 60: *P* < 0.05 with aSV = 80 and 100, *P* < 0.001 with aSV = 120; and BF = 50 vs BF = 60: ns with all aSV

**Fig. 7:**
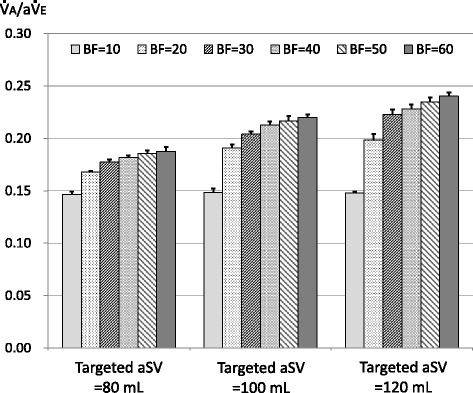
Ventilation efficiency ($\dot{\mathrm{V}}$A/a$\dot{\mathrm{V}}$E) measured with targeted actual stroke volume (aSV) of 80, 100, and 120 mL. Legend: Bar graph indicates mean $\dot{\mathrm{V}}$A/a$\dot{\mathrm{V}}$E (*n* = 5), and vertical bar indicates standard deviation. The results of statistical significance test are as follows: BF = 10 vs BF = 20–60: *P* < 0.001 with all aSV; BF = 20 vs BF = 30–60: *P* < 0.001 with all aSV; BF = 30 vs BF = 40: ns with aSV = 80 and 120, *P* < 0.01 with aSV = 100; BF = 30 vs BF = 50: *P* < 0.001 with aSV = 80 and 100, *P* < 0.01 with aSV = 120; BF = 30 vs BF = 60: *P* < 0.001 with all aSV; BF = 40 vs BF = 50: ns with all aSV; BF = 40 vs BF = 60: *P* < 0.05 with aSV = 80 and 100, *P* < 0.01 with aSV = 120; and BF = 50 vs BF = 60: ns with all aSV

aSVs with the target of 80, 100, and 120 mL were not significantly different at respective BF rates (Additional file 2). Figure 6 shows the $\dot{\mathrm{V}}$A, and Fig. 7 shows the $\dot{\mathrm{V}}$A/a$\dot{\mathrm{V}}$E with the targeted aSV of 80, 100, and 120 mL. In all targeted aSVs, the $\dot{\mathrm{V}}$A and the $\dot{\mathrm{V}}$A/a$\dot{\mathrm{V}}$E with a BF of 20 L/min or more were significantly higher than those with a BF of 10 L/min (*P* < 0.001), and those with a BF of 30 L/min or more were significantly higher than those with a BF of 20 L/min (*P* < 0.001). In all targeted aSVs, the $\dot{\mathrm{V}}$A and the $\dot{\mathrm{V}}$A/a$\dot{\mathrm{V}}$E with a BF of 50 or 60 L/min were significantly higher than those with a BF of 30 L/min (the *P* value was different for the targeted aSV with a BF of 50 L/min, and *P* < 0.001 with a BF of 60 L/min).

**Fig. 8:**
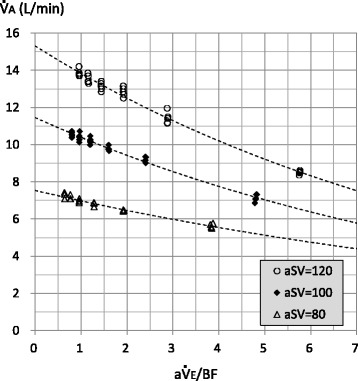
Relationship between actual minute volume divided by bias flow (a$\dot{\mathrm{V}}$E/BF) and alveolar ventilation ($\dot{\mathrm{V}}$A). Legend: Dotted curves are the exponential approximations. The coefficient of correlations (*R*) and *P* values are as follows: aSV = 80: *R* = − 0.982, *P* < 0.001; SV = 100: *R* = − 0.991, *P* < 0.001; and aSV = 120: *R* = − 0.992, *P* < 0.001. The *Y*-intercepts are 7.54 (aSV = 80), 11.46 (aSV = 100), and 15.31 (aSV = 120)

Figure 8 shows the relationships between a$\dot{\mathrm{V}}$E/BF and $\dot{\mathrm{V}}$A with respective targeted aSVs (individual data). The $\dot{\mathrm{V}}$A was correlated to the exponential of the a$\dot{\mathrm{V}}$E/BF with all targeted aSVs. The *Y*-intercepts were 7.54 with a targeted aSV of 80 mL, 11.46 with a targeted aSV of 100 mL, and 15.31 with a targeted aSV of 120 mL.

## Discussion

We investigated the effect of the BF rate on alveolar ventilation and ventilation efficiency using an original lung model in the adult oscillator R100, and our study showed that increasing the BF rate improved the ventilation efficiency up to 30 L/min or more with a frequency of 8 Hz. This result is almost consistent with the previous large animal study [1], though the used frequency, oscillator, and the structure of the circuit were different from our study. In Japan, the R100 oscillator has been often used with the frequency around 8 Hz or more. In the OSCAR trial which used the R100 oscillator, the mean frequency was 7.8 Hz on the first day [9]. We therefore performed the experiments with a frequency of 8 Hz. The initial setting of the BF rate was 20 L/min, and there was no description of BF change in the management protocol in the OSCAR trial [9]. If the BF rate is inadequate, the resultant higher aSV might tend to impair the benefit of HFOV. In the animal study which showed the superiority of higher frequency for lung protection, the aSV was lower with higher frequency [10]. In their study, it was unclear whether the higher frequency or the lower aSV was more important. However, it might be conceivable that the lower aSV might be beneficial in HFOV as well as in conventional lung protective ventilation [11]. The improvement of ventilation efficiency might be a future direction for reaching the goal of HFOV.

In the previous studies that measured aSV with the 3100B oscillator, a pneumotachometer or a hot-wire anemometer was used with a data sampling rate of 1000 Hz [12, 13]. Iguchi et al. measured aSV with two adult oscillators (3100B and R100) using a position sensor placed on the lung model with a data sampling rate of 667 Hz [14]. Although the aSV measurements were steady and stable in our study, the data sampling rate of 200 Hz might not be adequate for the frequency of 8 Hz. Therefore, we averaged the 60 data points for 1 min. However, a higher sampling rate would be desirable for robust reliability of the aSV measurement.

We have used a 20-L plastic container as a lung model [15, 16], and we improved it by adding a simulated bronchial tree in this study. An advantage of a lung model study is that a wide range of sSVs can be evaluated. However, the simulated bronchial tree in our lung model was quite different from a real lung, with the airway resistance being low and the VD was rather small for an adult. Therefore, the obtained values of $\dot{\mathrm{V}}$A and $\dot{\mathrm{V}}$A/a$\dot{\mathrm{V}}$E cannot be applied in clinical situations, and the interpretation of the current results may be difficult. For example, the $\dot{\mathrm{V}}$A would be approximately 21 L/min with a frequency of 8 Hz, a sSV of 205 mL (maximum), and a BF of 20 L/min in Fig. 3. These settings might be almost equal to the mean setting values of the first day in the OSCAR trial (7.8 Hz and 213 mL) [9]. On the other hand, because the phenomenon that occurs in the lung model must be almost identical with the same aSV and the same frequency, the observed effect of the BF rate in our study must have been caused by different phenomena that occurred in the oscillator circuit. Therefore, it is conceivable that the effect of the BF rate on the ventilation efficiency has a similar tendency in clinical situations.

In clinical or experimental settings, the $\dot{\mathrm{V}}$A during HFOV had been determined as frequency^*a*^ × SV^*b*^ (the values for *a* and *b* were approximately 1 and 2, respectively) [17]. Therefore, we applied the power approximation curves to the relationships between sSV or aSV and $\dot{\mathrm{V}}$A, and those were well fitted (Figs. 3 and 4). This finding would indicate that our lung model reproduced the particular ventilation mechanism of HFOV to some extent. Although the number of the power was less than two possibly because of the incomplete bronchial tree model, the number of the power was affected by the BF rate (Figs. 3 and 4). We used $\dot{\mathrm{V}}$A/a$\dot{\mathrm{V}}$E as an index of ventilation efficiency in the same way as conventional ventilation, and it was proportional to the aSV (Fig. 5). However, increasing aSV did not increase the ventilation efficiency with the BF of 10 L/min (Figs. 5 and 7). Theoretically, the $\dot{\mathrm{V}}$A/a$\dot{\mathrm{V}}$E must be very low, especially in the range of aSV far less than VD, and it must increase with higher aSV, especially in the range of aSV far more than VD, because of the correlation of direct alveolar ventilation (i.e., convection). However, “CO_2_ washout” from the oscillator circuit would be considerably involved in this issue as discussed in the next paragraph.

In the adult oscillator, the oscillation unit is located on the inspiratory circuit side. Therefore, the major part of the exhaled gas would be pulled out to the inspiratory circuit during the expiratory phase of the oscillation. This has been described as “retrograde CO_2_ entrainment” in the 3100B oscillator [2], and some CO_2_ rebreathing could naturally occur [1]. Although some gas regurgitation from the expiratory circuit to the inspiratory circuit could occur during the expiratory phase of the oscillation, major gas regurgitation would be prevented by the one-way valve placed at the end of the expiratory circuit in the R100 oscillator. The exhaled gas would mix with the fresh gas in the inspiratory circuit, and the CO_2_ would be diluted. Then, the mixed gas would go to the lung (aSV) and to the expiratory circuit (BF/cycle) during the next inspiratory phase of the oscillation. The former would include CO_2_ rebreathing, and the latter would be “CO_2_ washout.” The dilution of exhaled CO_2_ by the gas mixing in the inspiratory circuit would basically be affected by the ratio of the aSV and the BF rate per cycle (BF/cycle), that is, the ratio of the a$\dot{\mathrm{V}}$E and the BF rate per minute. Based on this concept, we examined the relationship between $\dot{\mathrm{V}}$A and a$\dot{\mathrm{V}}$E/BF (Fig. 8). Exponential curve fitting was used so that the *X*-intercept would be infinite because the $\dot{\mathrm{V}}$A would be zero without the BF. Although we performed the experiments with a single frequency (8 Hz), Fig. 8 would suggest that increasing the aSV or increasing the frequency would increase the need for a BF rate. The former could be a factor that affected the relationship between the aSV and $\dot{\mathrm{V}}$A/a$\dot{\mathrm{V}}$E (Fig. 5), as discussed in the paragraph above. Increasing aSV would promote the ventilation phenomena occurring in the lung model, although the need for BF would increase. Then, increasing aSV would induce a relative insufficiency of BF because of the constant BF rate in our study. In the case with the BF of 10 L/min, a$\dot{\mathrm{V}}$E/BF is 3.84, 4.8, and 5.76 with the aSV of 80, 100, and 120 mL, respectively. To keep an a$\dot{\mathrm{V}}$E/BF of 3.84, BF rates of 12.5 and 15 L/min are necessary with the aSV of 100 and 120 mL, respectively. This would be a reason why increasing aSV did not increase the ventilation efficiency with the BF of 10 L/min (Figs. 5 and 7). All results of our study could be influenced by this issue and must be carefully interpreted. Turner et al. reported that increasing the BF rate had little effect on the ventilation using an infant oscillator (3100A) with BF rates of 10, 20, 30, and 40 L/min [7], although we must consider the fact that they used a juvenile swine (mean body weight 15.1 kg) and a relatively low aSV would be used. On the other hand, because it is conceivable that CO_2_ rebreathing does not occur with an infinite BF rate, the *Y*-intercepts in Fig. 8 would indicate the upper limits of the improvement of $\dot{\mathrm{V}}$A by increasing the BF rate.

The gas mixing that occurred in the inspiratory circuit could not be completed. Conceptually, the BF might be separated from the gas that completely mixes with the exhaled gas (effective BF) and the gas that does not mix with the exhaled gas (ineffective BF). The mean concentration of CO_2_ exhausted from the expiratory circuit in the steady state must be $\dot{\mathrm{V}}$CO_2_/BF. In the case that all BFs are effective BFs, the mean concentration of rebreathing CO_2_ must be equal to this value. Namely, mean rebreathing PCO_2_ would be 15.2, 7.6, 5.1, and 3.8 mmHg with BFs of 10, 20, 30, and 40 L/min, respectively, when the $\dot{\mathrm{V}}$CO_2_ is 200 mL/min. If some BFs are ineffective BFs, rebreathing PCO_2_ might be higher. Although the ratio of effective BF is unclear, this concept may help us to understand the mechanism of “CO_2_ washout” by the BF.

## Conclusions

In this lung model study, ventilation efficiency improved at a BF equal to or more than 30 L/min compared to a BF equal to or less than 20 L/min with the R100 oscillator. The mechanism of improving ventilation efficiency by increasing the BF rate would be the decreased CO_2_ rebreathing, and the need for a BF rate would depend on a$\dot{\mathrm{V}}$E.

## Additional files

Additional file 1: Relationship between setting stroke volume (sSV) and airway pressure amplitude (AMP) measured at the Y-piece. Legend: Markers indicate the sSV and mean AMP (*n* = 5). Standard deviation is not indicated. The results of the statistical significance test are as follows: BF = 10 vs BF = 20: *P* < 0.001 with sSV = 80–110 and 160–180, *P* < 0.05 with sSV = 120, ns with sSV = 130–150; BF = 10 or 20 vs BF = 30 or 40: *P* < 0.001 with all sSV; and BF = 30 vs BF = 40: ns with sSV = 80, *P* < 0.001 with sSV = 90–180. Dotted curves are second-order approximation curves. Coefficient of correlations (*R*) and *P* values are as follows: BF = 10: *R* = 0.992, *P* < 0.001; BF = 20: *R* = 0.997, *P* < 0.001; BF = 30: *R* = 0.995, *P* < 0.001; and BF = 40: *R* = 0.990, *P* < 0.001. (DOCX 55 kb)

Additional file 2: Actual stroke volume (aSV) with targeted aSVs of 80, 100, and 120 mL. Legend: The bar graph indicates the mean aSV (*n* = 5), and the vertical bar indicates the standard deviation. There are no significant differences between BF from 10 to 60 L/min with all targeted aSVs. (DOCX 75 kb)

## Abbreviations

$$ \dot{\mathrm{V}} $$A: Alveolar ventilation
$\dot{\mathrm{V}}$A/a$\dot{\mathrm{V}}$E: Ratio of alveolar ventilation to actual minute ventilation
a$\dot{\mathrm{V}}$E: Actual minute ventilation
a$\dot{\mathrm{V}}$E/BF: Ratio of actual minute ventilation to bias flow rate
AMP: Airway pressure amplitude
ARDS: Acute respiratory distress syndrome
aSV: Actual stroke volume
BF: Bias flow
$\dot{\mathrm{V}}$CO_2_: Minute volume of insufflated CO_2_
CO_2_: Carbon dioxide
ETT: Endotracheal tube
HFOV: High-frequency oscillatory ventilation
MAP: Mean airway pressure
PCO_2_: Partial pressure of CO_2_
RCT: Randomized controlled trial
sSV: Setting stroke volume
SV: Stroke volume
VD: Dead space volume

## References

[1] Lunkenheimer PP, Redmann K, Stroh N, Gleich C, Krebs S, Scheld HH, Dietl KH, Fischer S, Whimster WF (1994). High-frequency oscillation in an adult porcine model. Crit Care Med.

[2] Bostick AW, Naworol GA, Britton TJ, Ori TR, French SK, Derdak S (2012). Inspiratory limb carbon dioxide entrainment during high-frequency oscillatory ventilation; characterization in a mechanical test lung and swine model. Respir Care.

[3] Fort P, Farmer C, Westeman J, Johannigman J, Beninati W, Dolan S, Derdak S (1997). High-frequency oscillatory ventilation for adult respiratory distress syndrome—a pilot study. Crit Care Med.

[4] Derdak S, Mehta S, Stewart T, Rogers M, Buchman TG, Carlin B, Lowson S, and the Multicenter Oscillatory Ventilation for Acute Respiratory Distress Syndrome Trial (MOAT) Study Investigators (2002). High-frequency oscillatory ventilation for acute respiratory distress syndrome in adults. A randomized, controlled trial. Am J Respir Crit Care Med.

[5] Fessler HE, Derdak S, Ferguson ND, Hager DN, Kacmarek RM, Thompson T, Brower RG (2007). A protocol for high-frequency oscillatory ventilation in adults: results from a round-table discussion. Crit Care Med.

[6] Solway J, Gaveriely N, Slutsky AS, Rossing TH, Drinker P, Saari AF, Drazen JM (1985). Effect of bias flow rate on gas transport during high-frequency oscillatory ventilation. Respir Physiol.

[7] Turner DA, Adams DF, Gentile MA, Lee W, Quick GA, Smith B, Cheifetz IM (2012). Bias flow does not affect ventilation during high frequency oscillatory ventilation in a pediatric animal model of acute lung injury. Pediatric Crit Care Med.

[8] Murray JF, Matthay MA, Luce JM, Flick MR (1988). An expanded definition of the adult respiratory distress syndrome. Am Rev Respir Dis.

[9] Young D, Lamb SE, Shah S, MacKenzie I, Tunnicliffe W, Lall R, Rowan K, Cuthbertson BH, OSCAR Study Group (2013). High-frequency oscillation for acute respiratory distress syndrome. New Engl J Med.

[10] Liu S, Yi Y, Wang M, Chen Q, Huang Y, Liu L, Xie J, Zhou D, Qiu H (2013). Higher frequency ventilation attenuates lung injury during high-frequency oscillatory ventilation in sheep models of acute respiratory distress syndrome. Anesthesiology.

[11] Brower RG, Matthay MA, Morris A, Schoenfeld D, Thompson BT, Wheeler A, Acute Respiratory Distress Syndrome Network (2000). Ventilation with lower tidal volumes as compared with traditional tidal volumes for acute lung injury and the acute respiratory distress syndrome. N Engl J Med.

[12] Sedeek KA, Takeuchi M, Suchodolski K, Kacmarek RM (2003). Determinants of tidal volume during high-frequency oscillation. Crit Care Med.

[13] Hager DN, Fessler HE, Kaczka DW, Shanholtz CB, Fuld MK, Simon BA, Brower RG (2007). Tidal volume delivery during high-oscillatory ventilation in adults with acute respiratory distress syndrome. Crit Care Med.

[14] Iguchi N, Hirao O, Uchiyama A, Mashimo T, Nishimura M, Fujino Y (2010). Evaluation of performance of two high-frequency oscillatory ventilators using a model lung with a position sensor. J Anesth.

[15] Shiba N, Nagano O, Hirayama T, Ichiba S, Ujike Y (2012). Humidification of base flow gas during adult high-frequency oscillatory ventilation: an experimental study using a lung model. Acta Med Okayama.

[16] Hirayama T, Nagano O, Shiba N, Yumoto T, Sato K, Terado M, Ugawa T, Ichiba S, Ujike Y (2014). Mean lung pressure during adult high-frequency oscillatory ventilation: an experimental study using a lung model. Acta Med Okayama.

[17] Pillow JJ (2005). High-frequency oscillatory ventilation: mechanisms of gas exchange and lung mechanics. Crit Care Med.

