# Supplementary material for: Bias flow rate and ventilation efficiency during adult high-frequency oscillatory ventilation: a lung model study
Source: Intensive Care Med Exp. 2018 Apr 19;6:11. doi: 10.1186/s40635-018-0176-3 (PMC5908780; doi:10.1186/s40635-018-0176-3)

Additional file 2

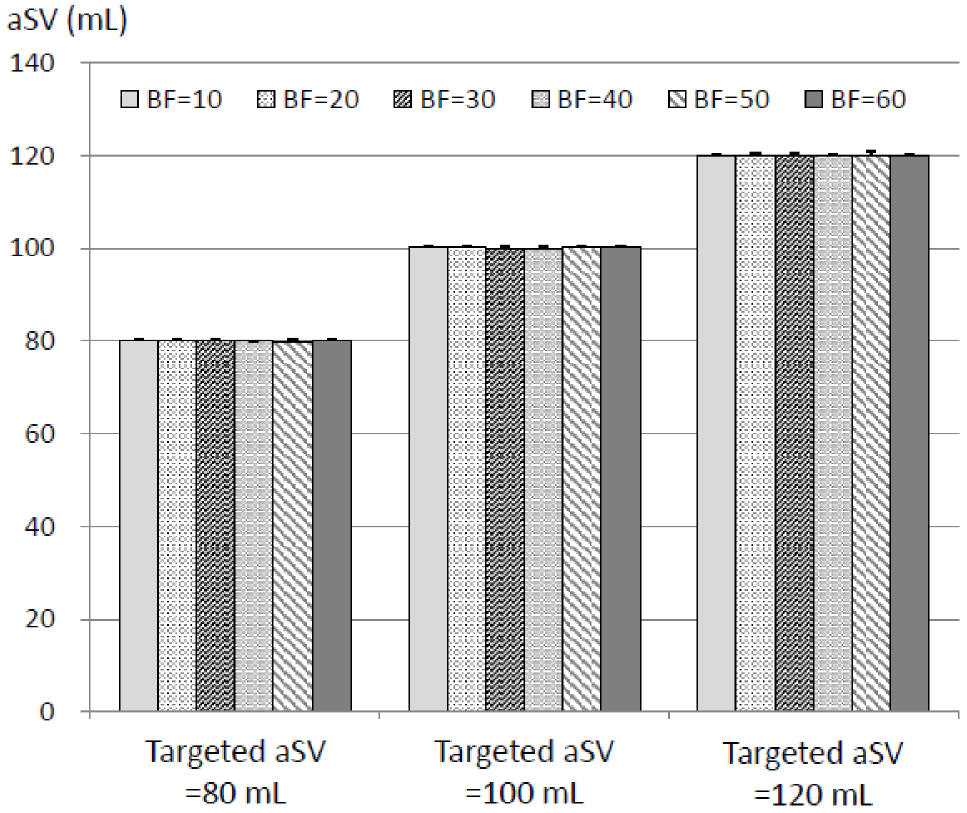

Supplement: Supplementary file 2 [file 40635_2018_176_MOESM2_ESM.docx]
